# Protocol for a randomized controlled trial on the impact of a health action process approach model-based mobile health intervention via WeChat on health behaviors of brucellosis patients

**DOI:** 10.3389/fpubh.2025.1619608

**Published:** 2025-11-12

**Authors:** Jing Wang, Yang Jiang, Zhenjie Yu, Jiawei Chen

**Affiliations:** 1 School of Public Health, Peking University, Beijing, China; 2 Jitang College, North China University of Science and Technology, Tangshan, Hebei, China; 3 Department of Infectious Diseases and Public Health, Jockey Club College of Veterinary Medicine and Life Sciences, City University of Hong Kong, Kowloon, Hong Kong SAR, China; 4 The Fourth Central Hospital of Baoding City, Baoding, Hebei, China

**Keywords:** brucellosis, health action process approach, randomized controlled trial, mobile health, health management

## Abstract

**Background:**

Brucellosis is a common zoonotic disease and a significant public health concern. Effective health management is essential for improving the self-management behaviors of brucellosis patients. This study aims to assess the effectiveness of a mobile health (mHealth) intervention based on the Health Action Process Approach (HAPA) model in enhancing self-management among brucellosis patients.

**Methods:**

This single-center, single-blind randomized controlled trial will be conducted at the Fourth Central Hospital of Baoding City, Hebei Province, China (ChiCTR2300071152). A total of 58 participants will be randomly assigned to the mHealth intervention group or the routine education group. Inclusion criteria: (1) Aged ≥18 years, (2) Diagnosed with brucellosis, (3) Familiar with WeChat, (4) Voluntary consent. Exclusion criteria include severe comorbidities, psychotropic medication use, and pregnancy. The intervention will be delivered via WeChat, using educational materials such as articles, videos, and case studies. The control group will receive standard discharge education. Primary outcomes include health behavior formation and health literacy, and secondary outcomes include psychological variables and behavior change. Statistical analyses will be conducted using SPSS Statistics 26.0. Data will be expressed as mean ± standard deviation or count (%). Parametric or non-parametric tests will be used as appropriate, and missing data will be handled using multiple imputation methods. A *p*-value <0.05 will be considered statistically significant.

**Discussion:**

This study will contribute to understanding the application of the HAPA model in guiding health interventions for brucellosis patients. It will be the first study in China to apply the HAPA model in this context, providing insights into its effectiveness for improving health behaviors and long-term outcomes in brucellosis patients.

## 1 Introduction

Brucella is a widespread zoonotic infection globally (1, 2). According to the World Health Organization (WHO), Brucella is defined as a disease that can be naturally transmitted from vertebrates to humans (3). It primarily originates from infected or severely infected animals, such as cows and sheep (4, 5). The main transmission routes include the digestive tract, skin, mucous membranes, and respiratory tract, through contact with blood, body fluids, and aerosols. The disease typically manifests with high fever, excessive sweating, malaise, and arthralgia, and can be more severe in some cases (6, 7). Brucella was first reported in China in 1905. In recent years, however, the prevalence of brucellosis has significantly increased (8, 9). National surveillance data reveal that, with the increase in livestock farming in China in recent years, the incidence rate of human brucellosis has shown a sustained upward trend in some regions. From 2004 to 2014, the national incidence rate rose from 0.92 to 4.2 cases per 100,000 people, and between 2004 and 2021, it increased by a total of 8.20%, seriously affecting both livestock production and public health (10–12).

The impact of brucellosis is twofold: it not only affects livestock production, causing substantial economic losses, especially in rural areas of developing countries where livestock farming is the primary economic activity (13–15), but also poses serious threats to human health and safety. One of the most effective ways to manage brucellosis is through public education on preventive behaviors, particularly for at-risk groups (15, 16). Health management strategies play a crucial role in controlling brucellosis (17). Therefore, educating individuals and changing their behaviors and lifestyles to prevent the disease is regarded as the best and most practical approach to controlling brucellosis.

Diagnosis and treatment after infection have limited effectiveness in controlling the disease. With advancements in medical technology, there is a growing demand for personalized and precise disease management for brucellosis patients. However, due to limitations in manpower and resources, primary healthcare organizations and medical staff often provide inadequate intervention management for brucellosis patients. Therefore, innovative mHealth interventions are essential for enhancing the management of brucellosis patients (18).

Mobile health, or mHealth, refers to the use of mobile devices, such as smartphones, patient monitoring devices, and wireless devices, to provide medical support and manage patient health (19). The rapid development of mobile networks has resulted in the widespread use of mobile electronic devices. A 2015 report indicated that more than seven billion mobile device subscriptions exist worldwide (20). This proliferation has made mHealth feasible, widespread, and replicable, utilizing features such as text messaging, email, phone calls, and mobile applications (21–24). One of the advantages of mHealth is that it requires no additional resources for implementation (25). The cost of monitoring clinical data and communicating educational information between doctors and patients is lower than that of face-to-face services (25). Furthermore, mHealth overcomes geographical barriers, enhances accessibility, and delivers health services to remote and underserved areas (26). By incorporating multimedia and big data, mHealth applications can enhance the health behaviors of individuals using these technologies (27, 28). MHealth lies at the intersection of communication technologies and personalized healthcare (29).

In the field of infectious disease management, mHealth interventions have shown significant potential. For instance, studies have shown that mHealth interventions, such as text message reminders and health information push notifications through social media platforms, can significantly improve adherence to antiretroviral therapy and improve patients’ mental health (30). Similar interventions have also been successful in tuberculosis management, particularly in enhancing patients’ self-management capabilities and treatment completion rates (31). These studies indicate that mHealth plays a critical role in improving health behaviors and self-management, especially in resource-poor regions and populations. However, mHealth-based interventions alone may lack theoretical guidance, potentially limiting the effectiveness of the intervention. Previous studies have emphasized that the design of mHealth interventions should be based on one or more theoretical models or frameworks to ensure their effectiveness and sustainability (32, 33).

**Figure 1:**
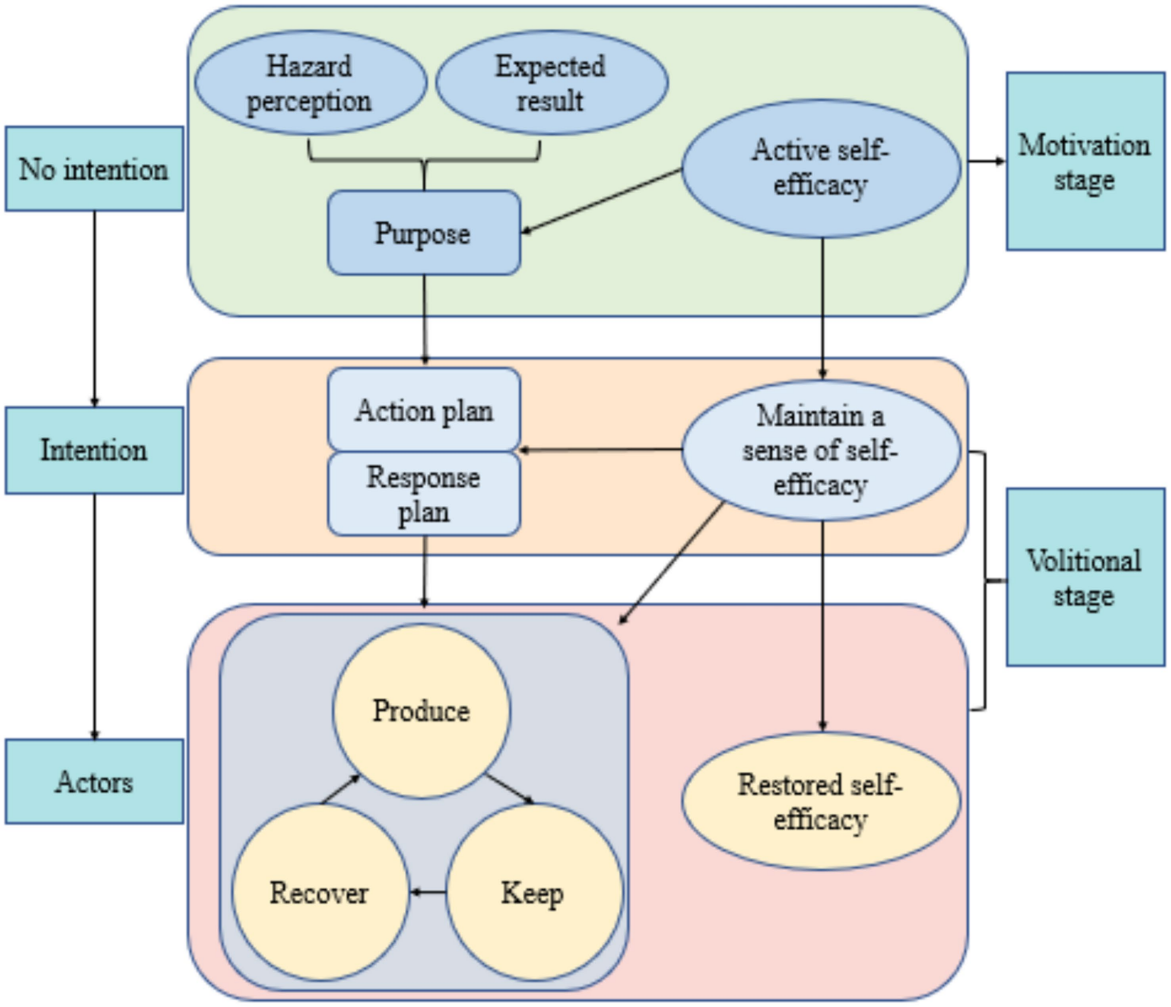
Schematic diagram of health action process approach.

The Health Action Process Approach (HAPA), shown in Figure 1 and developed by Litt et al. (34), suggests that health behavior change involves a series of psychological factors. It proposes that behavior change progresses through different stages, facilitating the effective transition from behavioral intentions to external behaviors. The HAPA model integrates relevant variables from the continuum model and stage theory model. It incorporates intrinsic motivational and volitional factors, such as self-efficacy, risk perception, and outcome expectancy (35), as well as behavioral intentions and action plans (36). It also considers extrinsic behavioral activities, such as inaction, irregularity, and regularity, as well as macro-environmental factors that affect the formation of intentions, initiation of actions, and maintenance of behaviors. By classifying individuals into different groups based on their motivational, volitional, and behavioral factors, the HAPA model links behavioral intentions to health behaviors through the use of implementation intentions, such as action plans, coping plans, self-monitoring, and self-regulation, thereby promoting the effective transition from intentions to behaviors (37). HAPA-based interventions have been proven effective in promoting health behavior changes (38–42). Specifically, the HAPA model has been applied to the study of COVID-19-related health behaviors during the pandemic, where the motivational and volitional factors included in HAPA increased adherence to protocols such as wearing masks and handwashing (43, 44). Additionally, the HAPA model has been used to assess social distancing behaviors during the pandemic, further emphasizing its applicability (45). In managing chronic diseases, a HAPA-based study in Iran demonstrated that the seven structures of HAPA were effective in promoting a healthy diet in patients with type 2 diabetes (46).

To date, no research has focused on the application of the HAPA model in the health management of brucellosis patients. Therefore, applying the HAPA model in the health management of brucellosis patients could significantly contribute to improving their health behaviors.

This study aims to develop a health education intervention model for brucellosis patients based on the HAPA framework. The first step involves assessing the current level of knowledge regarding brucellosis among patients. Drawing from best practice evidence, brucellosis-related information will be integrated to design a personalized health management intervention program for patients. The intervention will be delivered through the WeChat platform and mobile health devices, utilizing doctor-patient interaction for interactive health education. The goal is to deliver lifestyle-based behavioral interventions to assist patients in developing self-management habits.

The intervention tested in this trial will be based on the Health Action Process Approach (HAPA) model, which incorporates behavior change processes such as self-efficacy, risk perception, and the formation of action plans. This theoretical framework will guide the development and delivery of mHealth-based interventions, ensuring that the intervention is rooted in behavioral science to improve health outcomes in brucellosis patients. By integrating multimedia content and offering continuous support through mobile health devices, the intervention aims to enhance patient engagement and improve long-term self-management behaviors. The control group will receive usual care, which does not include mHealth interventions based on behavior change models. Usual care for brucellosis patients typically involves standard medical treatment, such as general health education, but lacks structured mHealth behavioral interventions. The choice of usual care as the comparator is justified by the absence of structured mHealth behavioral interventions for brucellosis. Since no established mHealth interventions are available for brucellosis, the comparison with usual care, which does not include a behavior change intervention, is the most appropriate to evaluate the effectiveness of the proposed intervention.

A rational research design will be used to analyze the impact of mobile health devices on the behavioral changes of brucellosis patients and the underlying mechanisms. This study aims to provide a clinical basis for the treatment plan, prognosis, and rehabilitation of brucellosis patients, serving as an initial step toward the broader implementation of mobile health interventions. Furthermore, the findings of this intervention may inform policy recommendations and guide technical transformations in the development of mobile health management applications.

The specific research objectives of this study include the following:

(1) To develop and evaluate an mHealth-based intervention model, integrating the HAPA model, aimed at improving health behaviors and enhancing self-management in brucellosis patients.
(2) To compare the effectiveness of the HAPA-based mHealth intervention with traditional management models, hypothesizing that the mHealth intervention will more effectively improve health behaviors and enhance self-management.

## 2 Methods

### 2.1 Study design

**Figure 2:**
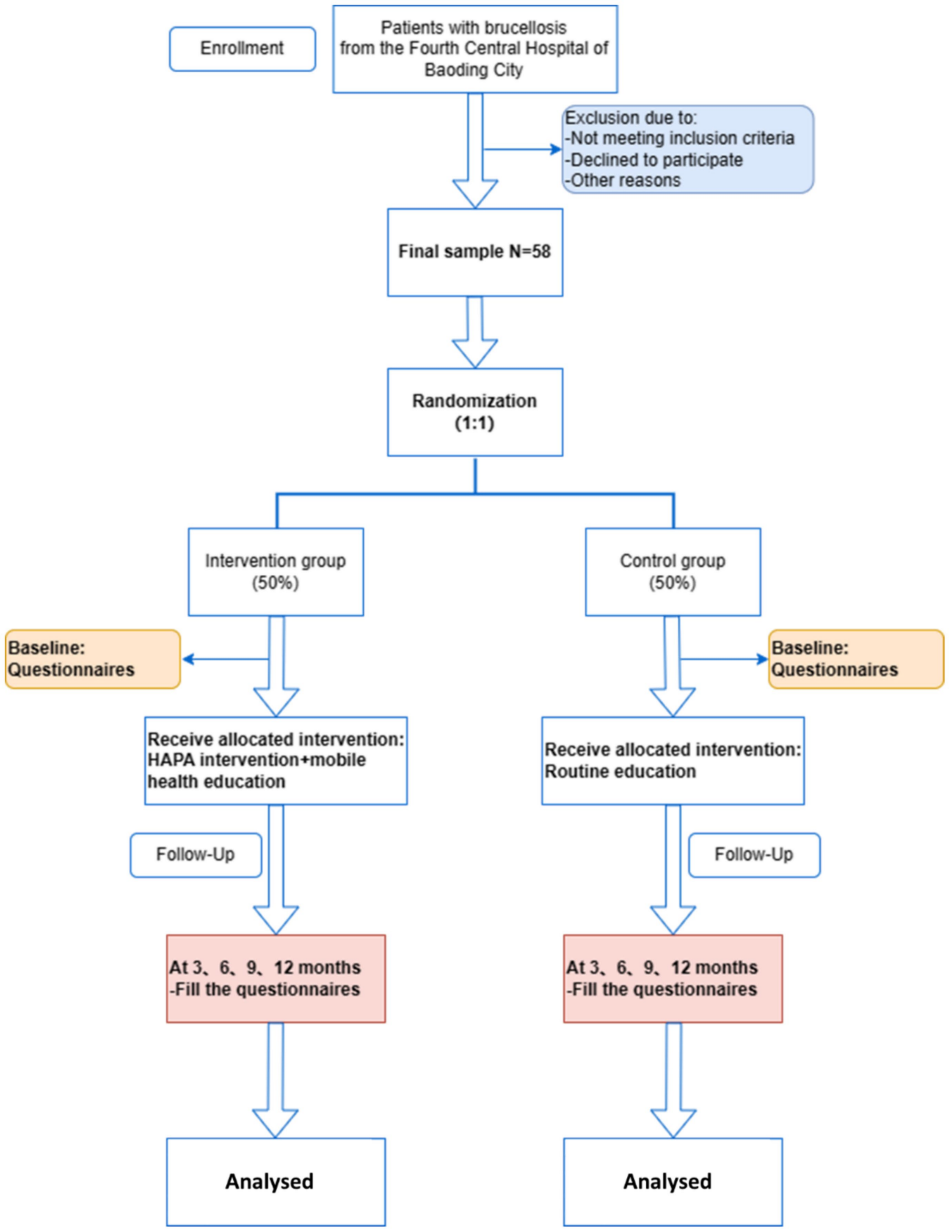
Flow chart of patient recruitment and study implement.

The study is an intervention randomized controlled trial in Baoding No. 4 Central Hospital, Hebei Province. The study employs quantitative research methods, using questionnaires based on brucellosis-related knowledge and internationally recognized scales for measurement. This approach ensures the objectivity of the study. The study will be conducted from December 2024 to December 2025. The intervention will last for 3 months, followed by a 12-month follow-up phase for data collection and evaluation. The questionnaire was given at the beginning then every 3 months which was then analyzed. The control group will receive conventional education, while the experimental group will receive mHealth education. The study aims to compare the effectiveness of mHealth interventions on the health behaviors of brucellosis patients based on the HAPA model, hypothesizing that mHealth interventions are more effective than conventional interventions (43). The flow chart of the study is shown in Figure 2 and Table 1.

**Table 1:** Timeline for study enrollment, intervention, and evaluation.

	Study period						
	Recruitment	Allocation	Intervention		Follow-up		
Timepoint	-T1	0	Month 0	Month 3	Month 6	Month 9	Month 12
Enrollment							
Informed consent	√						
Eligibility screen	√						
Allocation		√					
Intervention							
HAPA-mHealth			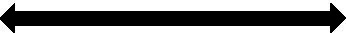				
Control			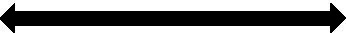				
Assessments							
Sociodemographic variables			√				
Proportion of individuals adopting health behaviors			√	√	√	√	√
Knowing rate of health knowledge			√	√	√	√	√
Refill and medication adherence scales			√	√	√	√	√
Quality of life			√	√	√	√	√
Depression and anxiety			√	√	√	√	√
Perceived stress scale			√	√	√	√	√
Perceived social support			√	√	√	√	√
Satisfaction				√	√	√	√

### 2.2 Study setting, randomization and blinding

The study will be a single-center, single-blind randomized controlled trial conducted at the Fourth Central Hospital of Baoding City, Hebei Province. Convenience sampling will be employed to recruit patients diagnosed with brucellosis. In this study, patient enrollment was conducted in batches to ensure feasibility, sample balance, and control of potential confounding factors. Specifically, eligible patients were enrolled in batches based on established time points, screening criteria, and enrollment procedures to ensure the study’s orderly and efficient progress.

Randomization was performed using Excel software to ensure unbiased group allocation. A new worksheet was created with numbers 1 to *N* in column A, where *N* represents the total number of participants. In column B, the formula = RAND() was used to generate random numbers between 0 and 1 for each participant. These random numbers were then fixed by copying and pasting them as ‘values’ to prevent modifications. The participants were then sorted by their random numbers in ascending order, and group assignments were made accordingly. The random allocation sequence will be generated by the researchers.

A single-blind design was implemented to ensure blinding. Participants were blinded to their group allocation, while evaluators remained aware of the group assignments. Allocation concealment was achieved using the envelope method. Adequate opaque envelopes were prepared, each labeled with a unique identifier number. A small slip of paper containing group assignment information was placed inside each envelope and sealed. After enrollment, participants received envelopes in sequential order based on their numerical identifiers and opened them independently to reveal their group assignment. The distribution and opening of envelopes were supervised by a designated individual to ensure the integrity of allocation concealment, and the process was documented for transparency. Unused envelopes were securely stored to preserve allocation concealment and maintain the single-blind design.

### 2.3 Study participants

The study was approved by the Ethics Committee of Baoding No. 4 Central Hospital. Participants will be patients from Baoding No. 4 Central Hospital, located in Baoding City, Hebei Province, China. Recruitment of study participants is expected to begin in December 2024. After enrolling in the trial and signing the informed consent form through face-to-face communication at the hospital, each participant will begin the formal trial intervention. Therefore, participants will be recruited and begin the intervention at different times. They will all complete the 3-month intervention cycle.

#### 2.3.1 Inclusion criteria

(1) Patients aged ≥18 years
(2) The patient is a permanent resident of Hebei Province (annual time away from home is less than 1 month).
(3) All patients meet the relevant diagnostic criteria for brucellosis in the Brucella Disease Diagnostic and Treatment Guidelines and the diagnosis of brucellosis was confirmed by pathological examination (47);
(4) The patient’s daily use of a smartphone and familiarity with general WeChat functions;
(5) Patients voluntarily agree to participate in the study by signing the informed consent form.

#### 2.3.2 Exclusion criteria

(1) The patient has no fixed contact information, no family member to act as a contact, and cannot be easily reached by phone or WeChat.
(2) The patient is currently taking psychotropic medication.
(3) The patient exhibits mental symptoms, such as confusion, slurred speech, or uncooperativeness
(4) The patient has serious comorbidities or physical impairments.
(5) The patient has participated in other clinical trials
(6) Pregnant or breastfeeding women
(7) Any other reasons deemed unsuitable for participation in the trial.

### 2.4 Sample size

This study is a randomized controlled trial with two groups: the mobile health intervention group and the conventional education group. The primary outcome is the health behavior formation rate among the participants. Based on a literature review (48), the compliance rate for behavior formation in the mobile health intervention group is expected to be 90%, compared to 60% in the control group. The significance level is set at *α* = 0.05, with a 90% confidence level. Using PASS15 software, the sample size for the intervention group was calculated as N1 = 23, and for the control group, N2 = 23. Considering a 20% follow-up loss rate, at least 29 participants are required in each group, resulting in a total of 58 participants.

### 2.5 Intervention

There will be two groups involved in this study, as follow.

#### 2.5.1 Mobile health intervention group

Health education in the mobile health intervention group will be delivered via the WeChat platform on smartphones. The physician or nurse will use WeChat’s messaging function to communicate with the patient promptly. The physician or nurse will address the patient’s concerns promptly. The physician or nurse can also intervene in patient care promptly through the WeChat platform (49).

To ensure high-quality delivery of the intervention, the professionals involved in delivering health education through WeChat (physicians and nurses) will need to meet the following criteria:

- Physicians must hold a valid medical degree in general practice or a relevant specialty and have at least 2 years of experience in patient care.
- Nurses must be licensed and have at least 1 year of experience in patient education or health promotion.
- All professionals must undergo a brief training program on the HAPA model, the principles of mobile health interventions, and effective communication through digital platforms like WeChat.

##### 2.5.1.1 Pre-intentional stage

At this stage, patients have not yet developed a willingness to improve health behaviors or engage in functional exercises.

###### 2.5.1.1.1 Action self-efficacy

(1) Purpose: to help patients develop initial self-efficacy and motivation to change their health behaviors.
(2) Measures: each patient will receive a booklet titled “Health Education for Patients with Brucellosis.” While distributing the booklet, the physician will explain common issues, encourage a positive approach to the disease, and guide patients to actively improve their health behaviors (31). During the initial consultation, physicians will add patients’ WeChat contact information and categorize them for follow-up health education. For patients with low health awareness, physicians will promptly discuss reasons for the lack of awareness, help identify solutions, and encourage mutual communication between patients and physicians.

###### 2.5.1.1.2 Expected outcomes

(1) Purpose: to provide patients with an initial understanding of brucellosis and its later stages.
(2) Measures: physicians will provide one-on-one re-education to patients via WeChat, based on a self-designed push program and the brochure “Health Education for Brucellosis Patients” (50). The push program includes scientific articles on brucellosis-related concepts, disease processes, and case studies. The focus is on the negative outcomes of advanced brucellosis and the benefits of effective disease management. The program also emphasizes the importance and effectiveness of adopting healthy behaviors for better outcomes.

###### 2.5.1.1.3 Risk perception

(1) Purpose: to enhance patients’ risk perception and foster a willingness to engage in positive health behaviors.
(2) Measures: physicians will push scientific articles on the disease process to patients via WeChat, based on the “Health Education for Brucellosis Patients” manual and a self-designed push program. The push program includes case studies to emphasize the dangers of brucellosis and its potential adverse outcomes (51). Additionally, the importance of forming healthy behaviors and their effectiveness in improving outcomes was emphasized. For patients with low health beliefs, the emphasis will be on the harmful effects of brucellosis on productivity and the benefits of controlling the epidemic.

##### 2.5.1.2 Intent stage

###### 2.5.1.2.1 Action plan

(1) Purpose: enhance patients’ self-efficacy for maintenance
(2) Measures: patients will receive 10-min health guidance from the physician via WeChat every Monday. The physician will help patients develop a long-term health behavior improvement plan and provide daily reminders via WeChat. The content of health education topics will be based on multimedia, in the form of articles, pictures, or videos sent to the advance WeChat group. For patients with low adherence, physicians will emphasize the dangers of neglecting animal husbandry hygiene, the risks of infectious diseases, and the benefits of maintaining hygienic habits and adhering to medication schedules. For patients with moderate adherence, physicians will focus on praise and encouragement to help maintain their confidence. For patients with high adherence, physicians will focus on monitoring their progress. Physicians will proactively seek recovery progress from all patients, guide them on the correct use of medication, and encourage active cooperation in treatment. The physician will encourage all patients to fill out the “Health Behavior Diary,” documenting daily health behaviors, issues, and experiences encountered. Patients will also be encouraged to communicate with the physician promptly, and their health behavior progress will be regularly evaluated.

###### 2.5.1.2.2 Response plan

(1) Purpose: to maintain patient self-efficacy by addressing challenges encountered during treatment.
(2) Measures: healthcare workers are essential in guiding patients who are livestock farmers towards successful recovery. Patients are encouraged to prioritize their recovery and are informed about the risks associated with their condition. Additionally, their families will be educated to increase awareness of the disease. Tailoring healthcare guidance to patients’ lifestyle habits is essential. Furthermore, collaborating with and seeking the supervision of their families is crucial. Addressing irregular medication intake is critical, as healthcare workers engage both patients and their families in discussions. Analyzing the reasons for inconsistent medication practices allows healthcare workers to encourage and acknowledge patients’ efforts to adopt healthier behaviors. In cases where patients show negative emotions and stop treatment-related health behaviors, promptly analyzing the reasons is essential. Appropriate strategies should be developed to help patients self-regulate their emotions, facilitating a swift resumption of health behaviors if interrupted (52).

#### 2.5.2 Control group

In the control group, patients will receive the standard discharge education provided upon their discharge from the hospital. As part of this education process, patients will be given a “Brucellosis Health Education” brochure, which contains essential information about brucellosis, including lifestyle recommendations, dietary guidelines, medication instructions, and potential adverse reactions to be aware of. Following discharge, patients will receive a telephone follow-up within a specified period, and an additional follow-up will take place 3 months post-discharge (53). These follow-ups are intended to assess patient progress, adherence to instructions, and any emerging concerns related to their condition.

#### 2.5.3 Regulations on participation adjustment for interventions

During the implementation of the health education program, participants retain the right to voluntarily withdraw from the study at any time. The research team will suspend or modify intervention measures under the following circumstances:

(1) Participant-initiated withdrawal due to personal reasons;
(2) Termination requested by the participant or legal guardian based on health or privacy concerns;
(3) Emergence of health-related unexpected issues during the intervention (e.g., sudden illness or exercise-related injuries);
(4) Research team-determined suspension based on safety evaluations.

All withdrawal requests and intervention adjustments must be fully documented in case report forms, including reasons for withdrawal, communication details, follow-up measures, and strict adherence to ethical review requirements for participant privacy protection.

### 2.6 Plans to promote participant retention and complete follow-up

To promote participant retention and ensure complete follow-up, the following strategies will be implemented:

(1) Regular reminders: participants will receive regular reminders via phone calls, text messages, or emails about upcoming visits and follow-up appointments.
(2) Telephone contact: in addition to scheduled visits, the research team will maintain regular telephone contact with participants to address any concerns, remind them about the study requirements, and ensure continued participation.
(3) Incentives: participants will be offered small incentives (e.g., transportation reimbursement or gift cards) to encourage retention and timely follow-up.
(4) Data collection for dropouts: if participants discontinue or deviate from the intervention protocol, efforts will be made to collect their data, including interviews and questionnaires, to minimize bias and understand reasons for discontinuation.

### 2.7 Collection and screening of health education materials

#### 2.7.1 Sources of health education materials

The health education materials for this study will be sourced from both domestic and international databases, such as WeChat, Wikipedia, doctor Q&A platforms, and ZhiNET, and will include both video and graphic formats. The educational materials, including medical records, will be scientifically accurate and sourced from Baidu documents or relevant literature with appropriate references.

#### 2.7.2 Screening of healthy materials

After the health education materials are collected and organized, experts in Brucella research will review the content according to predefined criteria. These criteria will include scientific accuracy, clarity, relevance to brucellosis management, and comprehensiveness. Experts will assess the materials for the following factors:

(1) *Scientific accuracy*: the materials must present information that is consistent with current scientific understanding and clinical guidelines on brucellosis.
(2) *Clarity and comprehensibility*: materials should be written clearly and be accessible to the target population, including individuals with varying health literacy levels.
(3) *Relevance*: the materials must address key aspects of brucellosis management, including disease awareness, prognosis, self-management strategies, and prevention methods.
(4) *Credibility*: sources must be reliable, such as peer-reviewed literature, trusted health organizations, or recognized medical professionals.
(5) *Multimedia appropriateness*: visual and video materials must be of high quality, informative, and easy to understand for patients.

After the review, experts will suggest necessary modifications to improve the content. Only high-quality materials meeting these criteria will be included in the study’s health education database. The materials will be categorized into sections such as disease awareness, prognosis, and other aspects of care. These materials will be used to create personalized push recommendations for patients. The push database will include article titles, relevant links, sources, material types, and other pertinent information (54).

### 2.8 Outcomes measure

Medical staff will distribute the questionnaire via the Questionnaire Star web platform. Patients will complete it online, receiving instructions either in person (during hospitalization) or by phone (after discharge). Demographic information will be collected through the case system and self-designed questionnaires (Table 1).

#### 2.8.1 Primary outcome

The primary outcome is the proportion of individuals adopting health behaviors and knowing rate of health knowledge.

##### 2.8.1.1 Proportion of individuals adopting health behaviors

The health behavior was assessed using a self-designed health behavior scale, which consists of five domains: (1) frequency of personal protective measures during contact with livestock (5 items), (2) frequency of personal protective measures when handling lambs and aborted materials (5 items), (3) frequency of personal protective measures when vaccinating livestock (5 items), (4) frequency of cleaning measures when cleaning animal pens (3 items), and (5) frequency of hand hygiene maintenance after contact with cattle, sheep, their placental tissues, or meat products (4 items). Each item was rated on a 5-point Likert scale: “Never (0),” “Rarely (1),” “Sometimes (2),” “Often (3),” and “Almost Always (4).” One reverse-scored item was included in each domain. The total score ranges from 0 to 110, with higher scores indicating better health behavior.

This scale was developed to evaluate the frequency of health behaviors related to occupational safety and hygiene practices among individuals at risk for brucellosis.

The proportion of individuals adopting health behaviors is calculated by determining the proportion of participants who meet the health behavior standard, as assessed by the health behavior questionnaire. A threshold score will be established to determine whether an individual has adopted health behaviors, with a score above 70 considered indicative of good health behavior formation. The adopting proportion is then calculated by dividing the number of individuals exceeding this threshold by the total number of participants.

##### 2.8.1.2 Knowing rate of health knowledge

The Brucellosis Health Knowledge Awareness questionnaire was designed based on the “National Brucellosis Surveillance Program” and tailored to the specific context of Baoding City, Hebei Province. The questionnaire includes items on awareness of brucellosis, its clinical symptoms, and modes of transmission. Participants indicated their knowledge, with correct answers marked as “aware” and incorrect or unknown answers as “unaware.”

The awareness rate for each item was calculated as the number of correct responses divided by the total number of valid respondents, multiplied by 100%. The overall knowledge awareness rate was calculated by dividing the total number of correct responses by the product of the total number of respondents and the total number of questions, then multiplying by 100%.

This questionnaire assesses health knowledge about brucellosis within the local population, providing essential information for health interventions.

#### 2.8.2 Secondary outcomes

##### 2.8.2.1 Refill and medication adherence scales

The scale has two dimensions: medication adherence and medication refilling (55). Medication adherence includes four items, while medication refilling includes three, for a total of seven items. Participants were scored on a 4-point Likert scale, ranging from “never” to “all the time.” The 7th question was a reverse question setting, with a total score of 7–28. A lower score indicates better adherence. The scale is as follows: Question 1: How often do you forget to take your medication? Question 2: How often do you decide not to take your medication? Question 3: How often do you forget to fill your prescription? Question 4: How often do you still need to take your medication but do not have it on hand? Question 5: How often do you miss taking your medication when you are feeling better? Question 6: How often do you miss taking your medication when you are not feeling well? Question 7: How often do you plan ahead and refill your medications before they run out? The ARMS is a valid and reliable self-report measure of medication adherence. The Chinese version of the ARMS scale demonstrates good internal consistency reliability (Cronbach’s *α* = 0.731) (56).

##### 2.8.2.2 Quality of life, EQ-5D-5L

The EQ-5D-5L is an enhanced version of the EQ-5D, adding five additional levels to the original five dimensions for a more refined health status assessment. The EQ-5D-5L scale consists of two parts, the five-dimensional description and the visual analogue score. The five-dimensional description includes five dimensions: mobility, self-care, daily activities, pain or discomfort, and anxiety or frustration. Each dimension has five levels of difficulty: no difficulty, little difficulty, moderate difficulty, great difficulty, and complete difficulty. The visual analogue score measures overall health on a scale from 0 to 100, with higher scores indicating better health. The visual analogue scale is a vertical scale, with a top score of 100 for “best health” and a bottom score of 0 for “worst health.” It provides a continuous variable for measuring overall quality of life. The Cronbach’s *α* coefficient of this scale is 0.857 (57).

##### 2.8.2.3 Anxiety depression ultra short form, PHQ-4

The PHQ-4 is a simplified version of the Anxiety and Depression Scale, with two dimensions: depression and anxiety. It consists of four entries, which are scored on a Likert 4 scale (1 = not at all, 2 = for a few days, 3 = for more than half of the days, and 4 = almost every day). Participants are required to assess their experiences of anxiety and depression using this scale. The total score ranges from 4 to 16, with higher scores indicating more severe anxiety and depression symptoms. This concise and easy to understand scale enables medical staff to quickly identify patients with anxiety and depression symptoms and provide an initial assessment. The PHQ-4 has a Cronbach’s *α* of 0.82, demonstrating good internal consistency (58).

##### 2.8.2.4 Perceived stress scale, PSS-4

The Perceived Stress Scale (PSS) is one of the most widely used tools in the world for measuring psychological stress. Developed by Cohen et al., the original version contained 14 entries (PSS-14), consisting of seven negatively descriptive and seven positively descriptive items. Instead of focusing on specific events, the PSS assesses the degree to which participants perceive their lives as unpredictable, uncontrollable, or overloaded. Each item is scored on a scale of 0–4, with higher scores reflecting greater perceived stress. To create shorter versions, Cohen et al. selected entries from the PSS-14, resulting in the PSS-10 and PSS-4, respectively. For the total scale score, scores of the positive items were reversed and all item scores were summed, with higher scores indicating higher perceived stress. In this study, the PSS-4 was used to evaluate stress trends, with a Cronbach’s *α* coefficient of 0.75 for the items (59).

##### 2.8.2.5 Perceived social support scale, PSSS-12

The scale developed by Zimet et al. (75) consists of three dimensions: family support, friend support, and other support. Each dimension contains four entries, making a total of 12 entries. Participants were rated on a 7-point Likert scale from “strongly disagree” to “strongly agree,” with scores ranging from 12 to 84. A higher total score indicates a higher level of perceived social support. The scale categorizes the level of perceived support into three categories: low (12–36 points), intermediate (37–60 points), and high (61–84 points). The original scale was initially used with American college students and has since been translated into multiple languages for widespread use in various countries. The translations have shown good reliability, with an internal consistency coefficient of 0.93 (59). Furthermore, the scale has been used in studies on perceived social support and psychological resilience in patients with ischemic stroke and coronary heart disease.

##### 2.8.2.6 Customer satisfaction questionnaire (CSQ-3)

The CSQ is a widely used questionnaire for assessing user satisfaction with telemedicine services. This is likely because the CSQ measures various attributes, including the physical environment, procedures, facilitators, service type, treatment staff, service volume or duration, service quality, outcomes, and overall satisfaction. The scale consists of three items, each rated from 1 (low satisfaction) to 4 (high satisfaction). Higher total scores indicate better satisfaction. The Cronbach’s alpha coefficient for the scale is 0.87, indicating good validity both overall and for each component (60).

### 2.9 Statistical analysis

Statistical analyses will be performed using SPSS Statistics 26.0 software. Count data will be expressed as the number of cases and rate (%), while measurement data will be presented as mean ± standard deviation. Before performing parametric tests, normality assumptions will be tested using the Shapiro–Wilk test. If data is normally distributed, parametric tests, such as the independent *t*-test, may be used. However, if the normality assumption is violated, non-parametric tests, such as the Mann–Whitney U test, will be used to compare changes in scale scores between baseline and endpoint between the two groups. The Wilcoxon signed-rank test will be employed for within-group comparisons of scale scores between baseline and endpoint. Demographic and clinical characteristics at baseline will be summarized descriptively for each group. Categorical variables will be reported as counts and percentages, and continuous variables as mean ± standard deviation.

For the primary analysis, all randomized participants will be included in an intention-to-treat (ITT) analysis, regardless of whether they complete the intervention. For secondary analyses, only participants who complete the intervention and follow-up will be included in a per-protocol (PP) analysis.

Regarding missing data, we will explore the missing data and compare the distribution of key variables. If the missing data is reasonable in amount and assumed to be Missing at Random (MAR), multiple imputation methods will be used to handle the missing data for both ITT and PP analyses. This ensures that the handling of missing data is consistent across both analysis groups. Baseline variables will be used as auxiliary variables for imputation, and multiple imputed datasets will be generated. Linear regression and logistic regression will be applied to analyze the imputed datasets, and the results will be pooled to provide final estimates and standard errors. A two-sided test will be used, and statistical significance will be defined as *p* < 0.05. The detailed statistical analysis plan for this study is available for public access at the Chinese Clinical Trial Registry: ChiCTR Statistical Analysis Plan (https://www.chictr.org.cn/showproj.html?proj=196659).

### 2.10 Study management

#### 2.10.1 Data management

##### 2.10.1.1 Data collection

All study results, including clinical and laboratory data, will be recorded in the participants’ medical records and case report forms (CRFs). The researchers are responsible for ensuring that all sections of the CRFs are accurately filled out, and that each piece of information can be verified against source data. Each completed CRF must be dated and signed. Data collection will be carried out by trained research staff, who will adhere to standard operating procedures (SOPs) to ensure the accuracy and consistency of the data. All data will be collected in strict accordance with the research protocol approved by the ethics committee. Participants’ personal information and data will be kept strictly confidential, and all records will use identification numbers instead of personal identifying information.

##### 2.10.1.2 Data storage and archiving

The researchers will store all trial data (including participant identification numbers, source data, and researcher documents) and related correspondence materials in the research database. All source data and related documents will be archived for the long term in accordance with applicable laws and regulations after the completion of the trial. Data will be stored in a controlled-access computer system, with access restricted to authorized personnel only, ensuring the confidentiality and integrity of the data. The data will be encrypted to prevent unauthorized access.

##### 2.10.1.3 Data management personnel

Data management will be handled by a dedicated research team and data management personnel. The data management team will be responsible for regularly checking data quality to ensure the integrity and accuracy of the data. Every individual involved in data management will receive training and sign confidentiality agreements to ensure adherence to ethical requirements and privacy protection standards. The principal investigator and data management personnel will jointly oversee the data management process, ensuring that data entry and storage comply with the trial protocol.

##### 2.10.1.4 Data disclosure and blinding

This study employs a single-blind design, where participants are unaware of their group assignments, while the researchers are informed. Prior to data analysis, the researchers will ensure that the blinding is maintained. All group allocation information will be provided to the relevant personnel in sealed envelopes after the completion of the study. Data analysis will be conducted by an independent statistical analysis team, and the team will not be involved with the group allocation information during data processing and analysis to ensure the impartiality of the results.

##### 2.10.1.5 Data analysis and safety

All data will be stored in a secure research database with strict access controls to ensure confidentiality and integrity. During the analysis phase, the data will undergo rigorous preprocessing and cleaning. Missing data will be handled using appropriate statistical methods. All analysis results will be processed using statistical software, and the researchers will follow the pre-established analysis plan. The final analysis results will be reviewed and verified by the statistical team to ensure data accuracy and reliability.

#### 2.10.2 Adverse event collection and evaluation

##### 2.10.2.1 Definition and collection of adverse events

Although this study involves a behavioral intervention, potential adverse effects or harm to participants must still be considered and monitored. Adverse events in this study include, but are not limited to, negative reactions potentially related to the intervention process, psychological stress, social impacts, or any unintended effects arising from participation.

While the study is considered low-risk, the research team will regularly monitor and collect data on any adverse events throughout the trial period. All participants will be informed that they can report any potential health or emotional adverse reactions at any time during the study. Such reports will be kept confidential, and participants will be encouraged to contact the research team immediately if they experience any negative effects.

Adverse event data will be collected through multiple channels, including face-to-face interviews, self-reported questionnaires, and routine follow-up visits. This comprehensive data collection method will ensure that all potential negative consequences are identified and addressed.

##### 2.10.2.2 Adverse event assessment and management

All reported adverse events will be assessed by the clinical team to determine whether they are related to the intervention and to classify their severity (mild, moderate, or severe). For each adverse event, the following details will be recorded: the time of occurrence, nature of the event, duration, outcome, and whether any medical intervention is required.

Serious adverse events (SAEs), defined as events requiring medical intervention or those that significantly impact the participant’s health, will be immediately reported to the ethics committee for review. Appropriate actions, including further medical evaluation and adjustments to the intervention if necessary, will be taken in consultation with the ethics committee and healthcare professionals involved in the study.

##### 2.10.2.3 Potential negative consequences of the intervention

Although the behavioral intervention is designed to improve health behaviors, there are potential risks of unequal allocation of healthcare resources or exacerbating social inequalities among certain groups of participants. The research team will pay particular attention to these socio-economic impacts during the analysis phase to ensure that no participant group is unfairly disadvantaged in terms of access to resources or benefits of the intervention. All participants will have equal opportunities for participation and the intervention will be implemented equitably across all groups.

If any participant group experiences unintended negative consequences due to socio-economic disparities, these will be addressed promptly, and the intervention will be adjusted as needed to ensure fairness and equal access for all participants.

#### 2.10.3 Ethics and regulations

##### 2.10.3.1 GCP (good clinical practice)

The procedures set out in this trial protocol relating to the conduct, evaluation and documentation of this trial are designed to ensure that all participants in the trial adhere to the ethical principles described in GCP and the current revision of the Declaration of Helsinki. The trial will be conducted in accordance with the requirements of local laws and regulations. The trail has been approved by the Ethics Committee of the Fourth Central Hospital of Baoding City.

##### 2.10.3.2 Informed consent

Prior to undergoing a clinical trial, subjects will be required to voluntarily agree to participate after an explanation of the nature, scope and possible consequences of the clinical trial. Subjects must give written consent. Informed consent will be obtained from each individual patient or the subject’s legally authorised representative. Patients who are unable to sign but are able to understand the significance of participating in the study may give verbally witnessed informed consent. These patients must clearly demonstrate that they are willing to participate voluntarily and must be able to understand the interpretation of the contents of the information sheet.

##### 2.10.3.3 Confidentiality

Prior approval has been obtained from the Ethics Committee before the commencement of this study.

Authorised consent for the use and/or disclosure of personal and/or health data must be obtained from the patient prior to enrolment. To protect patient privacy, patient’s age will be recorded on the CRF without recording the patient’s year of birth, and initials will be recorded on the CRF. The consistency of the data collected on the CRFs from the study centres will be checked and a data query form will be issued for inconsistent data requiring clarification by the doctor.

During clinical trials, results stored on computers will be stored in accordance with local data protection laws and will be handled in strict confidence. In order to protect this data, organisational procedures have been implemented to prevent the distribution of data to unauthorised persons. Appropriate provisions of local data legislation will be fully honoured. Authorised persons (clinical supervisors) may inspect subject-related data collected during the trial to ensure that the data are legally protected.

##### 2.10.3.4 Researcher responsibilities

Each investigator should ensure that all personnel assisting with the trial are fully informed about the protocol, any modifications to the protocol, the trial treatment, and their duties and functions related to the trial. Investigators should maintain a list of co-investigators and other appropriately qualified persons delegated with important judgement-related responsibilities.

##### 2.10.3.5 Approval of test programmes and amendments

Prior to the start of the trial, the trial protocol, informed consent document and any other appropriate documentation will be presented to the independent study team members. It must mention the date on which the decision was made and must be duly signed by a study member. All ethical and legal requirements must be met before the first subject is enrolled in the trial.

If any revisions to the study protocol are required, they will be led by the research team and submitted to the ethics committee for review and approval. After the revisions, all relevant parties (including the ethics committee, study team members, and participants) will be promptly provided with the updated protocol. Notifications of the revisions will be communicated through email and formal documentation, ensuring that all stakeholders are informed of and agree to the changes. The research team will maintain records of all revisions to ensure transparency and consistency of the protocol.

##### 2.10.3.6 Ethics committees

The trial will have to inform the Ethics Committee of all subsequent protocol revisions that require formal approval under local legal requirements. The Ethics Committee will have to be informed in advance of the trial procedures, if not described further in the documentation. The Ethics Committee will have to be informed of the end of the trial at the end of this trial. The results of this study will be made available to the public only after being published in a peer-reviewed journal.

#### 2.10.4 Clinical supervision

For quality assurance, the trial will comply with ethical and legal requirements and randomized clinical supervision will be provided in accordance with the IZKS Standard Operating Procedures based on the ICH-GCP guidelines. Supervisors will be carried out through personal visits that implement the clinical examination. Supervisors will review entries into the CRFs based on source documents. The investigator must allow the monitor to review all necessary documentation and must provide support to the monitor at all times.

### 2.11 Quality control

To ensure the integrity and reliability of the data throughout the study, several quality control measures will be implemented. First, double data entry will be employed, where two independent researchers will input the same data. Discrepancies will be flagged and resolved by the study team. Regular data audits will be conducted periodically (e.g., monthly) to check for data consistency, accuracy, and completeness. Any issues identified will be addressed promptly. All research staff will be trained on a standardized data collection protocol to ensure consistency in procedures.

Additionally, a Data Monitoring Committee (DMC) will oversee the data collection process and ensure that any issues related to outliers, missing values, or inconsistencies are addressed promptly. The DMC will be composed of [specific members, e.g., independent biostatisticians, ethicists, and clinical experts], and will operate independently from the study sponsor to avoid any conflicts of interest. Committee members will not have any financial or other interests that could influence their decision-making. The DMC will be governed by a formal charter that outlines its roles, responsibilities, and procedures for overseeing data integrity and compliance with the study protocol. The DMC is responsible for deciding whether the trial should be stopped, particularly if a pre-defined threshold for adverse events or harms is exceeded, in which case the study will be halted for safety reasons.

We will use an electronic data capture system to store all data, which includes built-in validation checks to minimize errors. Finally, quality assurance procedures will be implemented to monitor overall adherence to the study protocol, including informed consent and participant confidentiality. These measures will collectively ensure high-quality, reliable data for the study, minimizing errors and maintaining the integrity of the research findings.

### 2.12 Patient and public involvement

No patient or public involvement occurred in the design or conduct of this study. The study objectives, methods, and outcome measures were defined by the research team, without input from patients or the public.

## 3 Discussion

The use of mHealth in patient care has grown significantly since the COVID-19 outbreak, further accelerated by the World Health Organization’s call to expand the use of digital technologies to enhance remote care and support for patients (61). The rise of mHealth has proven to complement traditional clinical care effectively (62). Relevant studies have shown that mHealth plays a crucial role in improving patient adherence and enhancing acceptability, feasibility, and efficiency in brucellosis treatment (63). Brucellosis, a zoonotic disease endemic in the Middle East and present in regions such as China, Africa, and others (64), is classified as a re-emerging and neglected disease (65–68). In humans, the disease may cause mild symptoms, but it can also lead to more severe or chronic conditions. The disease is transmitted to humans through direct contact with tissues or blood from infected animals or through the consumption of contaminated animal products, including unpasteurized milk and cheese. Transmission via gas solvent may also occur (69–71). Although no human vaccine is currently available (72), efforts are underway to develop one (73). However, vaccines for animals are available. To prevent infection, individuals should avoid contact with infected animals or their products. Human behavior is critical in the control and prevention of brucellosis. Developing personal skills is based on the idea that individuals should be provided with the information and skills to take control of their health, develop strategies to combat the disease, and change norms to create a social environment that promotes health and prevention of brucellosis. Despite evidence suggesting that health promotion interventions grounded in behavioral science theories are more effective than those without such frameworks, few studies on brucellosis self-management have used theoretical models as a guide. Therefore, this study aims to evaluate an mHealth intervention based on the HAPA model to enhance self-management among individuals with brucellosis. Engaging in self-management behaviors is believed to influence both proximal health outcomes and distal outcomes of health status improvement (74). HAPA summarizes the key components of the health behavior change process and provides essential guidelines for intervention development, which is crucial for designing the interventions in this study. This study will be the first health intervention research in China to apply the HAPA theoretical model as a guide for managing brucellosis patients. The purpose of this study is to assess the effectiveness of the HAPA model as a theoretical guide for improving health behaviors in brucellosis patients and to validate its utility as an effective framework for guiding behavior change. This study will help address the research gap in postoperative health management for brucellosis patients in China, guided by health theory. To more comprehensively capture the impact of the intervention, the results and discussion will not only compare outcomes between the intervention and control groups, but will also include a longitudinal analysis across different time points during the study, enabling a more detailed understanding of behavioral changes over time.

The mode of transmission of brucellosis is relatively special, mainly through contact with animals or their excreta and ingestion of diseased animals to cause infection. This mode of transmission is not as intuitive and easy to identify as airborne or direct contact diseases, and is often overlooked as a result. Brucellosis has a long incubation period, typically between 2 weeks and 6 months, which makes it difficult to detect infection and implement effective preventive and control measures. Moreover, Brucella, a parthenogenetic intracellular parasite, is able to evade immune cell attacks and persist and multiply in the body. This characteristic renders the treatment of brucellosis challenging, often requiring long-term, combination medication, and is prone to relapse. Additionally, the clinical symptoms of brucellosis are complex and easily confused with other diseases, leading to misdiagnosis and mistreatment. This increases the importance of exploring ways to promote patients’ self-management to prevent disease recurrence and improve prognosis, particularly for patients in the livestock industry who are at a higher risk due to frequent contact with livestock. In recent years, the development of e-medicine has changed the way people seek medical advice. With the rapid advancement of mobile Internet technology, the internet has become a widely accessible channel for health information. Considering this, in combination with the current trend of mobile health interventions, relevant studies have shown the effectiveness of mobile health interventions in promoting health behaviors. In this study, the HAPA theoretical model will be used as a guiding framework to improve the intervention. This study will be the first in China to utilize mHealth interventions to promote self-management among Brucella patients. The incorporation of the HAPA theoretical model as a guiding framework increases the scientific rigor of the study design and fills a gap in the field of mHealth interventions utilizing the HAPA theoretical model. The results of this study will assess the feasibility and effectiveness of applying health-based theoretical models combined with mHealth applications in disease prognostic health management models. While this study aims to inform policy recommendations and technological applications for the development of mobile health management, these should be considered preliminary and will require additional evidence from larger trials to substantiate their broader applicability.

This study investigated the effectiveness of the mHealth intervention by comparing the experimental group with HAPA as the guiding theory of mHealth to the control group with routine discharge education. In future studies, it should also be compared with mHealth that is not based on the intervention theory. This comparison can provide insights into the impact of the theory-based approach on the effectiveness of the mHealth intervention.

The planned study, which aims to enroll 58 participants, serves as an initial exploratory investigation. However, its small sample size limits its ability to represent the full spectrum of brucellosis patients, particularly with regard to geographical, age, gender, and disease severity diversity. The study is further limited by being conducted at a single center, which raises concerns about the generalizability of the findings to other regions, particularly rural and underserved areas with differing healthcare access. Another limitation is the potential bias inherent in self-reported data, which may affect the accuracy of participants’ reported health behaviors and self-management practices, potentially introducing social desirability or recall bias. Furthermore, although a 12-month follow-up is planned, the long-term impact of the intervention remains uncertain, raising the crucial question of its durability and patients’ sustained adherence. The use of the WeChat platform for the mobile health intervention, although widespread in China, presents challenges as not all patients are familiar with or inclined to use this medium. Additionally, individual preferences for receiving health information vary, highlighting the need for further consideration in the study’s design and execution.

## References

[1] Ariza J Bosilkovski M Cascio A Colmenero JD Corbel MJ Falagas ME . Perspectives for the treatment of brucellosis in the 21st century: the Ioannina recommendations. PLoS Med. (2007) 4:e317. doi: 10.1371/journal.pmed.0040317, PMID: 18162038 PMC2222927

[2] Luo B Wang Q Yang S Song X Li Z. Epidemiological, clinical, and laboratory characteristics of 581 human brucellosis cases in Xinjiang, China. Front Microbiol. (2025) 16:1541277. doi: 10.3389/fmicb.2025.1541277, PMID: 40400681 PMC12092428

[3] Shakir R. Brucellosis. J Neurol Sci. (2021) 420:117280. doi: 10.1016/j.jns.2020.117280, PMID: 33358192

[4] Zhou S Qin H Shi Q Li S Chen J Chen Q. Changing patterns of epidemiological characteristics and spatial-temporal clusters of human brucellosis based on county level—China, 2011–2023. China CDC Wkly. (2025) 7:453–9. doi: 10.46234/ccdcw2025.075, PMID: 40376444 PMC12075457

[5] Mangalgi SS Sajjan AG Mohite ST Gajul S. Brucellosis in occupationally exposed groups. J Clin Diagn Res. (2016) 10:DC24–7. doi: 10.7860/JCDR/2016/15276.7673, PMID: 27190804 PMC4866102

[6] Moosazadeh M Nikaeen R Abedi G Kheradmand M Safiri S. Epidemiological and clinical features of people with Malta fever in Iran: a systematic review and Meta-analysis. Osong Public Health Res Perspect. (2016) 7:157–67. doi: 10.1016/j.phrp.2016.04.009, PMID: 27413646 PMC4927669

[7] Yang L Fan M Wang Y. Dynamic modeling of prevention and control of brucellosis in China: a systematic review. Transbound Emerg Dis. (2025) 2025:1393722. doi: 10.1155/tbed/1393722, PMID: 40302758 PMC12017112

[8] De Massis F Sacchini F Petrini A Bellucci F Perilli M Garofolo G . Canine brucellosis due to *Brucella canis*: description of the disease and control measures. Vet Ital. (2022) 58:5–23. doi: 10.12834/VetIt.2561.16874.1, PMID: 35766163

[9] Sibhat B Tessema TS Nile E Asmare K. Brucellosis in Ethiopia: a comprehensive review of literature from the year 2000-2020 and the way forward. Transbound Emerg Dis. (2022) 69:e1231–52. doi: 10.1111/tbed.14495, PMID: 35196417

[10] Majzoobi MM Hashmi SH Emami K Soltanian AR. Combination of doxycycline, streptomycin and hydroxychloroquine for short-course treatment of brucellosis: a single-blind randomized clinical trial. Infection. (2022) 50:1267–71. doi: 10.1007/s15010-022-01806-x, PMID: 35353333 PMC8966606

[11] Wen X Wang Y Shao Z. The spatiotemporal trend of human brucellosis in China and driving factors using interpretability analysis. Sci Rep. (2024) 14:4880. doi: 10.1038/s41598-024-55034-4, PMID: 38418566 PMC10901783

[12] Liu Z Wang M Shi Y Wang L Ren X Li Z . Epidemiological evolution profile of human brucellosis and socioeconomic factor correlation analysis—southern and northern areas, China, 1950-2021. China CDC Wkly. (2025) 7:460–6. doi: 10.46234/ccdcw2025.076, PMID: 40376445 PMC12075453

[13] Yu L Zheng H Wang Y Wang X Wang X. Spatiotemporal distribution and ecological factors of disease burden in Inner Mongolia's working age population with brucellosis from 2015 to 2020. Sci Rep. (2025) 15:13496. doi: 10.1038/s41598-025-96464-y, PMID: 40251217 PMC12008177

[14] Barua A Kumar A Thavaselvam D Mangalgi S Prakash A Tiwari S . Isolation & characterization of *Brucella melitensis* isolated from patients suspected for human brucellosis in India. Indian J Med Res. (2016) 143:652–8. doi: 10.4103/0971-5916.187115, PMID: 27488010 PMC4989840

[15] Ali S Akhter S Neubauer H Melzer F Khan I Abatih EN . Seroprevalence and risk factors associated with bovine brucellosis in the Potohar plateau, Pakistan. BMC Res Notes. (2017) 10:73. doi: 10.1186/s13104-017-2394-2, PMID: 28129787 PMC5273848

[16] Marvi A Asadi-Aliabadi M Darabi M Abedi G Siamian H Rostami-Maskopaee F. Trend analysis and affecting components of human brucellosis incidence during 2006 to 2016. Med Arch. (2018) 72:17–21. doi: 10.5455/medarh.2018.72.17-21, PMID: 29416212 PMC5789554

[17] Kansiime C Atuyambe LM Asiimwe BB Mugisha A Mugisha S Guma V . Community perceptions on integrating animal vaccination and health education by veterinary and public health workers in the prevention of brucellosis among pastoral communities of South Western Uganda. PLoS One. (2015) 10:e0132206. doi: 10.1371/journal.pone.0132206, PMID: 26218368 PMC4517904

[18] Rantala A Vuorinen AL Koivisto J Similä H Helve O Lahdenne P . A gamified mobile health intervention for children in day surgery care: protocol for a randomized controlled trial. Nurs Open. (2022) 9:1465–76. doi: 10.1002/nop2.1143, PMID: 34859602 PMC8859057

[19] World Health Organization. Global diffusion of eHealth: making universal health coverage achievable. Geneva: World Health Organization (2016).

[20] International Telecommunication Union. (2015). ICT facts & figures. Available online at: https://www.itu.int/en/ITU-D/Statistics/Documents/facts/ICTFactsFigures2015.pdf (accessed October 26, 2023)

[21] Gelano TF Assefa N Bacha YD Mahamed AA Roba KT Hambisa MT. Effect of mobile-health on maternal health care service utilization in eastern Ethiopia: study protocol for a randomized controlled trial. Trials. (2018) 19:102. doi: 10.1186/s13063-018-2446-5, PMID: 29433537 PMC5809837

[22] Ganasegeran K Renganathan P Rashid A Al-Dubai SA. The m-health revolution: exploring perceived benefits of WhatsApp use in clinical practice. Int J Med Inform. (2017) 97:145–51. doi: 10.1016/j.ijmedinf.2016.10.013, PMID: 27919374

[23] Androutsou T Kouris I Anastasiou A Pavlopoulos S Mostajeran F Bamiou DE . A smartphone application designed to engage the elderly in home-based rehabilitation. Front Digit Health. (2020) 2:15. doi: 10.3389/fdgth.2020.00015, PMID: 34713028 PMC8521815

[24] Zhang Y Fan S Hui H Zhang N Li J Liao L . Privacy protection for open sharing of psychiatric and behavioral research data: ethical considerations and recommendations. Alpha Psychiatry. (2025) 26:38759. doi: 10.31083/AP38759, PMID: 40110382 PMC11915712

[25] Wheeler TS Michael Vallis T Giacomantonio NB Abidi SR. Feasibility and usability of an ontology-based mobile intervention for patients with hypertension. Int J Med Inform. (2018) 119:8–16. doi: 10.1016/j.ijmedinf.2018.08.002, PMID: 30342690

[26] Weinstein RS Lopez AM Joseph BA Erps KA Holcomb M Barker GP . Telemedicine, telehealth, and mobile health applications that work: opportunities and barriers. Am J Med. (2014) 127:183–7. doi: 10.1016/j.amjmed.2013.09.032, PMID: 24384059

[27] Lv Z Chirivella J Gagliardo P. Bigdata oriented multimedia mobile health applications. J Med Syst. (2016) 40:120. doi: 10.1007/s10916-016-0475-8, PMID: 27020918

[28] Bhuyan SS Lu N Chandak A Kim H Wyant D Bhatt J . Use of Mobile health applications for health-seeking behavior among US adults. J Med Syst. (2016) 40:153. doi: 10.1007/s10916-016-0492-7, PMID: 27147516

[29] Cameron JD Ramaprasad A Syn T. An ontology of and roadmap for mHealth research. Int J Med Inform. (2017) 100:16–25. doi: 10.1016/j.ijmedinf.2017.01.007, PMID: 28241934

[30] Guo Y Xu Z Qiao J Hong YA Zhang H Zeng C . Development and feasibility testing of an mHealth (text message and WeChat) intervention to improve the medication adherence and quality of life of people living with HIV in China: pilot randomized controlled trial. JMIR Mhealth Uhealth. (2018) 6:e10274. doi: 10.2196/10274, PMID: 30181109 PMC6231726

[31] Bao Y Wang C Xu H Lai Y Yan Y Ma Y . Effects of an mHealth intervention for pulmonary tuberculosis self-management based on the integrated theory of health behavior change: randomized controlled trial. JMIR Public Health Surveill. (2022) 8:e34277. doi: 10.2196/34277, PMID: 35834302 PMC9335179

[32] Jiang Y Sun X Jiang M Min H Wang J Fu X . Corrigendum: impact of a mobile health intervention based on multi-theory model of health behavior change on self-management in patients with differentiated thyroid cancer: protocol for a randomized controlled trial. Front Public Health. (2024) 12:1414576. doi: 10.3389/fpubh.2024.1414576, PMID: 38741910 PMC11090245

[33] Sun X Jiang Y Wang J Fan S Fu X An Z . Effects of a mobile health intervention based on a multitheoretical model of health behavior change on anxiety and depression, fear of cancer progression, and quality of life in patients with differentiated thyroid cancer: a randomized controlled trial. BMC Med. (2024) 22:466. doi: 10.1186/s12916-024-03652-0, PMID: 39407174 PMC11475815

[34] Litt MD Kleppinger A Judge JO. Initiation and maintenance of exercise behavior in older women: predictors from the social learning model. J Behav Med. (2002) 25:83–97. doi: 10.1023/a:1013593819121, PMID: 11845560

[35] Tang C Raat H Yan M Zhang Q Li K Jiang M . Application of the health action process approach model for reducing excessive internet use behaviors among rural adolescents in China: a school-based intervention pilot study. BMC Public Health. (2021) 21:986. doi: 10.1186/s12889-021-10999-z, PMID: 34039318 PMC8152115

[36] Seeballuck C Blair A Donnelly J Towers A. Mobile apps for oral healthcare: recommendations for navigating uncharted terrain. Br Dent J. (2022) 233:462–6. doi: 10.1038/s41415-022-4971-6, PMID: 36151170

[37] van Nes KA van Loveren C Luteijn MF Slot DE. Health action process approach in oral health behaviour: target interventions, constructs and groups-a systematic review. Int J Dent Hyg. (2023) 21:59–76. doi: 10.1111/idh.12628, PMID: 36208281 PMC10092238

[38] Zhang CQ Zhang R Schwarzer R Hagger MS. A meta-analysis of the health action process approach. Health Psychol. (2019) 38:623–37. doi: 10.1037/hea0000728, PMID: 30973747

[39] Wu W Hu L Chen Y Cao F Ding S Wu T . Effectiveness of an online application of the health action process approach (HAPA) theory on oral hygiene intervention in young adults with fixed orthodontic appliances: a randomized controlled trial. BMC Oral Health. (2022) 22:192. doi: 10.1186/s12903-022-02219-w, PMID: 35590291 PMC9118762

[40] Asgari S Abbasi M Hamilton K Chen YP Griffiths MD Lin CY . A theory-based intervention to promote medication adherence in patients with rheumatoid arthritis: a randomized controlled trial. Clin Rheumatol. (2021) 40:101–11. doi: 10.1007/s10067-020-05224-y, PMID: 32588274 PMC7782392

[41] Scheerman JFM Hamilton K Sharif MO Lindmark U Pakpour AH. A theory-based intervention delivered by an online social media platform to promote oral health among Iranian adolescents: a cluster randomized controlled trial. Psychol Health. (2020) 35:449–66. doi: 10.1080/08870446.2019.1673895, PMID: 31621423

[42] Ahorsu DK Lin CY Imani V Carlbring P Nygårdh A Broström A . Testing an app-based intervention to improve insomnia in patients with epilepsy: a randomized controlled trial. Epilepsy Behav. (2020) 112:107371. doi: 10.1016/j.yebeh.2020.107371, PMID: 32861897

[43] Malik K Amir N Kusumawardhani AAAA Lukman PR Karnovinanda R Melisa L . Health action process approach (HAPA) as a framework to understand compliance issues with health protocols among people undergoing isolation at emergency hospital for COVID-19 Wisma Atlet Kemayoran and RSCM Kiara ultimate Jakarta Indonesia. Front Psych. (2022) 13:871448. doi: 10.3389/fpsyt.2022.871448, PMID: 35722553 PMC9199900

[44] Lao CK Li X Zhao N Gou M Zhou G. Using the health action process approach to predict facemask use and hand washing in the early stages of the COVID-19 pandemic in China. Curr Psychol. (2023) 42:6484–93. doi: 10.1007/s12144-021-01985-0, PMID: 34155429 PMC8210514

[45] Hamilton K Smith SR Keech JJ Moyers SA Hagger MS. Application of the health action process approach to social distancing behavior during COVID-19. Appl Psychol Health Well Being. (2020) 12:1244–69. doi: 10.1111/aphw.12231, PMID: 33006814 PMC7537318

[46] Ranjbaran S Shojaeizadeh D Dehdari T Yaseri M Shakibazadeh E. Using health action process approach to determine diet adherence among patients with type 2 diabetes. J Educ Health Promot. (2020) 9:170. doi: 10.4103/jehp.jehp_175_20, PMID: 32953901 PMC7482647

[47] Jiang H Feng L Lu J. Updated guidelines for the diagnosis of human brucellosis—China, 2019. China CDC Wkly. (2020) 2:487–9. doi: 10.46234/ccdcw2020.129, PMID: 34594685 PMC8393123

[48] Zong X-M Zhang H-H Li W-H Xue W-H . Brucellosis health education and behavior intervention effectiveness evaluation in Pingshan county. J Med Pest Control. (2015) 31:237–40.

[49] Jiang Y Liu F Guo J Sun P Chen Z Li J . Evaluating an intervention program using WeChat for patients with chronic obstructive pulmonary disease: randomized controlled trial. J Med Internet Res. (2020) 22:e17089. doi: 10.2196/17089, PMID: 32314971 PMC7201319

[50] Yeung AWK Tosevska A Klager E Eibensteiner F Tsagkaris C Parvanov ED . Medical and health-related misinformation on social media: bibliometric study of the scientific literature. J Med Internet Res. (2022) 24:e28152. doi: 10.2196/28152, PMID: 34951864 PMC8793917

[51] Hermesh B Rosenthal A Davidovitch N. The cycle of distrust in health policy and behavior: lessons learned from the Negev Bedouin. PLoS One. (2020) 15:e0237734. doi: 10.1371/journal.pone.0237734, PMID: 32817681 PMC7446867

[52] Dong Y Wang P Dai Z Liu K Jin Y Li A . Increased self-care activities and glycemic control rate in relation to health education via Wechat among diabetes patients: a randomized clinical trial. Medicine (Baltimore). (2018) 97:e13632. doi: 10.1097/MD.0000000000013632, PMID: 30558051 PMC6319995

[53] Wang W Zhang H. Behavior patterns and influencing factors: health information acquisition behavior of Chinese senior adults on WeChat. Heliyon. (2023) 9:e16431. doi: 10.1016/j.heliyon.2023.e16431, PMID: 37303534 PMC10248094

[54] Tse SY. Diabetes mellitus and periodontal disease: awareness and practice among doctors working in public general out-patient clinics in Kowloon west cluster of Hong Kong. BMC Fam Pract. (2018) 19:199. doi: 10.1186/s12875-018-0887-2, PMID: 30558542 PMC6297978

[55] Huang P Zheng-Dao D Sun BM Pan YX Zhang J Wang T . Bilateral anterior capsulotomy enhances medication compliance in patients with epilepsy and psychiatric comorbidities. CNS Neurosci Ther. (2019) 25:824–31. doi: 10.1111/cns.13118, PMID: 30868752 PMC6630004

[56] Wu J Tao Z Qian C Song Z Shen J. Optimization and validation of reliability and validity of continuation and medication adherence scale in elderly diabetic patients in China. J Pharmacoepidemiol. (2021) 30:706–12. doi: 10.19960/j.cnki.issn1005-0698.2021.10.012

[57] Keetharuth AD Hussain H Rowen D Wailoo A. Assessing the psychometric performance of EQ-5D-5L in dementia: a systematic review. Health Qual Life Outcomes. (2022) 20:139. doi: 10.1186/s12955-022-02036-3, PMID: 36171595 PMC9520934

[58] Löwe B Wahl I Rose M Spitzer C Glaesmer H Wingenfeld K . A 4-item measure of depression and anxiety: validation and standardization of the patient health Questionnaire-4 (PHQ-4) in the general population. J Affect Disord. (2010) 122:86–95. doi: 10.1016/j.jad.2009.06.019, PMID: 19616305

[59] Li L Peng T Liu R Jiang R Liang D Li X . Development of the psychosomatic symptom scale (PSSS) and assessment of its reliability and validity in general hospital patients in China. Gen Hosp Psychiatry. (2020) 64:1–8. doi: 10.1016/j.genhosppsych.2020.01.008, PMID: 32070913

[60] Ireland JL Boustead R Ireland CA. Coping style and psychological health among adolescent prisoners: a study of young and juvenile offenders. J Adolesc. (2005) 28:411–23. doi: 10.1016/j.adolescence.2004.11.002, PMID: 15925691

[61] WHO team. Global tuberculosis report. Geneva: World Health Organization (2021).

[62] Wang J Jiang Y Fu X Gou R Sun Z Li G . Evaluating the impact of interactive video-based case-based learning in clinical medical education: a randomized controlled trial. Front Med. (2025) 12:1556018. doi: 10.3389/fmed.2025.1556018, PMID: 40454152 PMC12123690

[63] Keutzer L Wicha SG Simonsson US. Mobile health apps for improvement of tuberculosis treatment: descriptive review. JMIR Mhealth Uhealth. (2020) 8:e17246. doi: 10.2196/17246, PMID: 32314977 PMC7201317

[64] Jiang H O'Callaghan D Ding JB. Brucellosis in China: history, progress and challenge. Infect Dis Poverty. (2020) 9:55. doi: 10.1186/s40249-020-00673-8, PMID: 32448394 PMC7247241

[65] Zhou K Wu B Pan H Paudyal N Jiang J Zhang L . One health approach to address zoonotic brucellosis: a spatiotemporal associations study between animals and humans. Front Vet Sci. (2020) 7:521. doi: 10.3389/fvets.2020.00521, PMID: 32984409 PMC7492289

[66] Zhang N Zhou H Huang DS Guan P. Brucellosis awareness and knowledge in communities worldwide: a systematic review and meta-analysis of 79 observational studies. PLoS Negl Trop Dis. (2019) 13:e0007366. doi: 10.1371/journal.pntd.0007366, PMID: 31048848 PMC6497230

[67] Bagheri Nejad R Krecek RC Khalaf OH Hailat N Arenas-Gamboa AM. Brucellosis in the Middle East: current situation and a pathway forward. PLoS Negl Trop Dis. (2020) 14:e0008071. doi: 10.1371/journal.pntd.0008071, PMID: 32437346 PMC7241688

[68] Ghanbari MK Gorji HA Behzadifar M Sanee N Mehedi N Bragazzi NL. One health approach to tackle brucellosis: a systematic review. Trop Med Health. (2020) 48:86. doi: 10.1186/s41182-020-00272-1, PMID: 33093792 PMC7574566

[69] Shojaei MS Tavakoly Sany SB Ghavami V Tehrani H. An educational intervention based on family-centered empowerment model to modify high-risk behaviors of brucellosis via mother education. Sci Rep. (2022) 12:18869. doi: 10.1038/s41598-022-23385-5, PMID: 36344585 PMC9640551

[70] Rasouli H Gharlipour Z Koohpaei A Mohamadbeigi A. Evaluating the effect of educational intervention on the modification of high-risk job behaviors in patients with brucellosis in rural areas of Arak County, Iran based on the family-centered empowerment model. Qom Univ Med Sci J (2023); 17:1619.1. Available online at: https://doaj.org/article/c241482988e0475ba8c537e7daf31709#:~:text=Given%20the%20complications%20of%20brucellosis%2C%20we%20sought%20to,with%20brucellosis%20in%20Arak%20County%2C%20Iran%2C%20in%202021

[71] de Figueiredo P Ficht TA Rice-Ficht A Rossetti CA Adams LG. Pathogenesis and immunobiology of brucellosis: review of *Brucella*–host interactions. Am J Pathol. (2015) 185:1505–17. doi: 10.1016/j.ajpath.2015.03.003, PMID: 25892682 PMC4450313

[72] Lalsiamthara J Lee JH. Development and trial of vaccines against *Brucella*. J Vet Sci. (2017) 18:281–90. doi: 10.4142/jvs.2017.18.S1.281, PMID: 28859268 PMC5583415

[73] Ziapour A Rezaei F Jafarzadeh M Mirzaei N Moradi-Asl E Nejhaddadgar N. Practical steps to design brucellosis preventive interventions: an intervention mapping approach. Health Sci Rep. (2025) 8:e70369. doi: 10.1002/hsr2.70369, PMID: 39867707 PMC11757818

[74] Ryan P Maierle D Csuka ME Thomson A Szabo A. Computer-based intervention to enhance self-management of calcium and vitamin D intake in women. West J Nurs Res. (2013) 35:986–1010. doi: 10.1177/0193945913483369, PMID: 23539320 PMC4545626

[75] Zimet GD Dahlem NW Zimet SG Farley GK. The multidimensional scale of perceived social support. J Personality Assess. (1988) 52:30–40. doi: 10.1207/s15327752jpa5201_2, PMID: 23539320

